# Seroprevalence and predictors of hepatitis A infection in Nigerian children

**DOI:** 10.11604/pamj.2015.20.120.5501

**Published:** 2015-02-12

**Authors:** Joanah Moses Ikobah, Henry Chima Okpara, Emmanuel Eyo Ekanem, Jacob Jackson Udo

**Affiliations:** 1 Department of Paediatrics, University of Calabar Teaching Hospital, Calabar, Cross River state, Nigeria; 2 Department of Chemical Pathology, University of Calabar Teaching Hospital, Calabar, Cross River State, Nigeria

**Keywords:** Hepatitis A virus, seroprevalence, predictors

## Abstract

**Introduction:**

Hepatitis A infection is prevalent in developing countries where sanitation is still a public health issue. In Nigeria, there is no epidemiological data on children for this infection. A community based study was carried out to establish the seroprevalence and predictors of this infection in children.

**Methods:**

A community based cross sectional study was carried out in Akpabuyo local Government Area of Cross River State in southern Nigeria. Multi-staged sampling technique was used to recruit 406 children aged 1-18 years. Blood samples were analysed for anti-HAV total antibody (IgM and IgG) using a commercial Enzyme-Linked Immunoassay Assay(ELISA). A multivariate logistic regression was used to identify factors that independently predicted the occurrence of anti-HAV total antibody. p value of < 0.05 was considered significant.

**Results:**

Two hundred and twenty four subjects tested positive for anti-HAV total antibody giving a prevalence rate of 55.2%. The median age for those positive was 9 years and for those without evidence of HAV infection was 4 years. One hundred and one (45.1%) males and 123 (54.9%) females were positive. The study population was mainly of the low social class with 94.1%. After multivariate analysis, predictors of HAV infection were age and social class.

**Conclusion:**

HAV infection was prevalent in the study population. Educational campaign is imperative and vaccine provision is advocated to further curb the spread of this infection.

## Introduction

Hepatitis A is a non-enveloped ribonucleic acid (RNA) virus that is transmitted faeco-orally [1–3]. It has a worldwide distribution with the highest prevalence in developing countries, where environmental and socio-economic conditions favour nearly universal exposure in early childhood [1–3]. Improvements in public health sanitation have led to a decline in the incidence of hepatitis A infections in the developed countries and to a shift of the time of first exposure to older age groups [4–6]. This is not so in the developing countries where sanitation is still a major public health issue and nearly all children are infected with HAV before the age of nine [5]. In most developed countries, endemic HAV transmission is unlikely [5]. In the developing regions of the world, inadequate sanitation results in continuous transmission of HAV infection in children and young individuals [4, 5]. Recent changes in the epidemiology of HAV infection and the availability of effective vaccines have renewed interest in this infection [4, 5].

There is significant difference in the seroprevalence of hepatitis A infection among children of different socioeconomic status with a lower prevalence of anti-HAV antibody among the higher socio-economic status and better environmental conditions [7, 8]. There is substantial underestimation of hepatitis A infections in developing countries because HAV infections in young children are mostly asymptomatic and therefore unrecognized. In preschoolers, HAV infection frequently causes acute liver failure [9–11]. In Nigeria, study done in the past on hepatitis A infection was an urban based hospital study involving children and adults [8]. This study was therefore designed to provide data on the first ever rural community based seroprevalence for Hepatitis A infection in healthy Nigerian children.

## Methods

**Study area:** The study was conducted in Akpabuyo Local Government Area (LGA) of Cross River State in south south geopolitical zone, Nigeria. Akpabuyo LGA is a suburb LGA bounded by Akamkpa LGA in the north, Calabar Municipality in the west, Bakassi LGA in the east and the Cross River in the south. It is made of 10 electoral wards with a total population of 313,097. The occupations of the residents include farming, trading, civil service and fishing.

**Design:** This was a community based cross sectional study to determine the seroprevalence and predictors of viral hepatitis A in children aged 1 to 18 years.

**Study period:** The study was carried out between April and June 2012

**Study population:** The study population consisted of children aged 1 to 18 years

**Ethical issues:** The study was approved by the Ethical Review Committee of University of Calabar Teaching Hospital and the Cross River State Health Research Ethics Committee. Informed consent was obtained from each parent / legal guardian of eligible participants prior to enrolment.

**Sampling technique:** Multi-stage sampling technique was used in this study and this involved three stages. The first stage was a simple random sampling technique used to select four out of 10 wards by balloting. In the second stage, proportionate sampling method was used to select 10 villages from the four selected wards while in the third stage, 40 children from alternate households in the selected villages were chosen from those eligible after a screening form was administered. Children who have been resident in Akpabuyo for less than one year were excluded from the study. A structured interviewer-administered questionnaire was then given to heads of the households.

**Data collection:** The following data were collected by using structured interviewer administered questionnaire: 1) General characteristics (age, sex); 2) family socioeconomic characteristics and sanitation: parents/guardian's occupation and education, total number of persons in the household, toilet types, method of disposal of domestic household waste, source of drinking water. The social class of parents/guardians was determined using the social classification proposed by Olusanya [12] considering the parents/guardian's occupation and educational qualification; 3) Clinical history was obtained from each subject to find out those who were eligible for the study.

**Laboratory investigations:** Two millitres (2mls) of venous blood was obtained from each eligible child under aseptic procedure into a clean plain bottle properly labeled with serial number tags. Samples collected were taken to the University of Calabar Teaching Hospital laboratory within one hour of collection and the serum was separated to be used for the analysis. The serum samples were stored at −20°C for a maximum period of five days before batch analysis. The sera were tested for anti-HAV total antibody by a competitive enzyme immunoassay (EIA) test with test kits from DRG International Inc., USA. Anti-HAV total antibody tested for both IgM and IgG. Test results were interpreted as a ratio of the absorbance of the sample (As) and the cut-off absorbance (Ac). The level of < 0.9mIU/mL for anti-HAV total antibody were considered negative; 0.9 to 1.1mIU/mL equivocal and >1.1mIU/mL positive. A negative result indicated that the subject has not been infected by HAV. Subjects with equivocal result were retested on a second sample taken two weeks later. A positive result was indicative of HAV infection.

### Statistical analysis

The data obtained were analyzed using statistical package for social sciences (SPSS) version 17.0 Inc. Chicago, Illinois – USA. Quantitative variables were summarized as median (Interquartile range IQR) and categorical data were summarized as frequency (percentage). Chi square was used to test for association between categorical variables. Likelihood-ratio chi square and Fishers exact test were applied where required. Multivariate logistic regression analysis was used to control for anticipated confounders. A p-value of < 0.05 was considered statistically significant. Results were presented in tables.

## Results

### General characteristics of the children

Four hundred and six children aged 1 - 18 years participated in this study. The age group 1 – 4 years was the highest represented with a total number of 150 (37.0%). The age group 15–18 years was least represented with a total of 51(12.6%). The median age was 6 years and the interquartile range was 3-12 years. Two hundred and seven (51.0%) were females and 199 (49.0%) were males, giving a female to male ratio of 1:1. Table 1 shows the age and sex distribution of the study population. Twenty four (5.9%) of the subjects belonged to the middle class and 382 (94.1%) were of the low social class. There was no subject in the high social class.

**Table 1:** Age and sex distribution of the study population

Age(years) Groups	Female n (%)	Male n (%)
1 - 4	62 (30.0)	88 (44.2)
5 - 9	58 (28.0)	51(25.6)
10 - 14	57(27.5)	39(19.6)
15 - 18	30(14.5)	21(10.6)
**Total**	207(100)	199(100)

### Seroprevalence of anti-HAV total antibodies

Two hundred and twenty four subjects tested positive for anti-HAV total antibody giving a prevalence rate of 55.2% (95% CI 42 – 53). No subject had equivocal result. The median age for those positive was 9 years with an inter quartile range of 5 – 13 years. The median age for those without evidence of HAV infection was 4 years with an interquartile range of 2 – 9 years. Thus those positive for anti –HAV total antibody were older than those without anti – HAV total antibody. This was statistically significant (p < 0.001). This is shown on Table 2.

**Table 2:** Prevalence of anti-HAV total antibody in relation to age of subjects

Age(Year) Group	Anti-HAV total antibody	
	Positive n (%)	Negative n (%)
1 – 4	53 (23.7)	97 (53.3)
5 – 9	65 (29.0)	44 (24.2)
10 – 14	68 (30.4)	28 (15.4)
15 – 18	38 (17.0)	13 (7.1)
**Total**	224 (100)	182 (100)

Table 3 shows distribution of anti-HAV total antibody by gender. One hundred and one (45.1%) males and 123 (54.9%) females were positive. This difference was not statistically significant (p = 0.080).

**Table 3:** Gender distribution for anti-HAV total antibody

Sex	Anti – HAV total antibody	
	Positive n (%)	Negative n (%)
Female	123 (54.9)	84 (46.2)
Male	101 (45.1)	98 (53.9)
**Total**	224 (100)	182 (100)

Table 4 shows the univariate and multivariate logistic regression for anti-HAV total antibody. At the univariate level, the associations of anti-HAV total antibody prevalence with age, duration of residence, number of persons in the household and social class were confirmed. The univariate logistic regression showed that for every one year increase in age, there was a 13% (95% CI 1.09-1.18) risk of being positive for HAV. Number of persons in the household in relation to HAV positivity showed that for every extra member of the household, there was a 9% (95% CI 1.01 – 1.17) increase risk of infection with hepatitis A.

**Table 4:** Logistic regression model for anti-HAV total antibody

	Univariate	Multivariate
	OR (95% CI) p-value	OR (95% CI) p-value
Age (years)	1.13 (1.09-1.18) < 0.001	1.12 (1.05 – 1.21) 0.002
Gender	0.70 (0.48 –1.04) 0.08	0.83 (0.54-1.27) 0.47
**Source of Water**		
Pipe borne	1	1
Others	1.18 (0.78-1.78) 0.43	1.04 (0.64-1.75) 0.94
**Duration of residence (years**)		
1– 5	1	1
5– 10	2.08 (1.27-3.41) 0.04	1.25 (0.70-2.24) 0.44
>10	3.56 (2.08-6.08) <0.001	0.95 (0.41-2.23) 0.91
Number of persons in household	1.09 (1.01-1.17) 0.02	1.01 (0.93-1.09) 0.83
**Faecal disposal method**		
Water cistern	1	1
Others	2.92 (0.54-1.27) 0.76	0.53 (0.24-1.14) 0.10
**Social class**		
Middle	1	1
low	3.19 (0.29-7.88) 0.01	6.59 (2.00-21.74) 0.002

At the multivariate level, age and social class remained significantly associated with having positive anti –HAV status after adjusting for other factors in the model. For every one year increase in age, the risk of developing HAV infection increased by 12% (95% CI 1.05 – 1.21) after adjusting for the influence of social class, gender, duration of residence, number of persons in the household and human waste disposal method. Also, those in the low social class had a 6.5 times increased risk of having HAV infection after adjusting for other factors in the model. Faecal disposal method, number of persons in the household, duration of residence, source of drinking water and gender did not significantly predict having HAV infection.

## Discussion

The seroprevalence of hepatitis A in this study was 55.2%. This prevalence was higher than that reported in the Ayoola's [8] study where he had a prevalence of 42.5% in those aged 5 – 19 years in Ibadan south-west Nigeria. The higher prevalence in this study could be due to the fact that this study was carried out in rural communities while Ayoola's [8] study was carried out in an urban hospital setting.

This study also showed that those positive for anti-HAV antibody were older than those without the infection. Ayoola [8], Jacobsen et al [13] and Colak et al [14] also demonstrated same phenomenon. The reason for this is that in endemic regions, HAV infection is acquired early in life [5]. The prevalence in the older age group is likely to be higher than the younger age because of cumulative effect.

There was no significant association between gender and seropositivity to anti-HAV antibody in this study. This was also demonstrated by Ayoola [8] and Gomes et al [15] This is possibly due to the fact that both sexes live in the same endemic environment and are exposed to the same predictors of the infection.

Duration of residence was significantly associated with positivity to anti-HAV antibody. To the best of the investigators’ knowledge this variable has not been assessed by other authors. The reason for this is probably that the longer a person stays in the environment, the higher the chances of exposure to the risk factors. This may also explain the association with increased age.

HAV infection is endemic in areas where sanitation is poor [5]. However method of faecal waste disposal, domestic waste disposal and source of drinking water were not significantly associated with the prevalence of anti-HAV antibody in this study. Escobedo-Melendez et al [16] working in Mexico also showed no association between sewage disposal method with prevalence of anti-HAV antibody. Vancelik et al [17] working in eastern Turkey, showed no significant association between source of drinking water and anti-HAV antibody. Sources of water they looked at were tap water and well water. In this study, though those who used bore hole water had the highest prevalence (63.83%), there was no significant difference in the prevalence of HAV between the sources of drinking water. This may reflect the generally poor quality of drinking water in the communities as no one used pipe borne water. In recognition of this possibility, Vancelik et al [17] has suggested that analysis of the quality of drinking water may be more important than just stating the source.

In this study, low social class was associated with increased prevalence of infection. Escobedo-Melendez et al [16] working in Mexico, reported significant association of low socioeconomic class with the presence of anti-HAV antibody. Gomes et al [15] working in Brazil demonstrated low level of parental education as a contributor to HAV antibody positivity in children. This could be due to reasons that those in the low social class could have poor hygiene and poor knowledge of the disease and its mode of transmission. This informs the need for educational campaign on the mode of transmission and prevention of this infection in the community.

Number of persons in the household was significantly associated with prevalence of anti-HAV antibody. Gomes et al [15] working on associated factors to seroprevalence of hepatitis A among school children in Brazil, similarly demonstrated that the higher the number of persons living in a household, the higher the risk of infection. This supports the possibility of person to person transmission of hepatitis A.

After multivariate analysis, age and social class remained significantly associated with having anti-HAV total antibody. For every one year increase in age, the risk of developing HAV infection increased by 12%. Gomes et al [15] showed, after multivariate analysis, that ages 11-14 years were associated with greater prevalence of IgG to HAV, indicating a progressive increase in positivity with age. Their study population age was between 7-14 years. In Gomes et al's [15] study, after multivariate analysis, the association between family income and prevalence of hepatitis A was no longer significant.

Gomes et al's [15] study was carried out in an area considered to have endemicity ranging from intermediate to high [5] unlike the communities where this study was carried out which has high endemicity of hepatitis A. High endemicity is found in countries with poor sanitary conditions and socially disadvantaged groups [5].

## Conclusion

More than half of the children aged 1 – 18 years in these communities were positive for Hepatitis A IgG and IgM antibodies. The environmental hygiene and social amenities are generally poor; therefore their effects on the seroprevalence could not be demonstrated. Health education campaign on the mode of transmission of the virus is imperative. In addition, HAV infection control could be used as an entry point for the provision of piped water, proper domestic and sewage disposal systems for these communities. Vaccine against hepatitis A is also advocated as a good step to control this infection. **Limitation of the study:** the kit used for this study tested for anti-HAV total antibody. It was therefore not possible to separate new infections from more prolonged ones.
